# P-143. Interpreting Prognostic Factors Associated with In-Hospital COVID-19 Mortality in a Peru Sample using Shapley Additive Explanations on Ensemble Learning

**DOI:** 10.1093/ofid/ofae631.348

**Published:** 2025-01-29

**Authors:** Na Dai, Ruben K Briceno, Alex Castaneda, Miguel Tresierra, Maribel Esteban, Moises Rosas, Rene Hinojosa

**Affiliations:** Michigan State University College of Osteopathic Medicine, Michigan; Michigan State University, East Lansing, Michigan; Universidad Cesar Vallejo, Trujillo, La Libertad, Peru; Universidad Cesar Vallejo, Trujillo, La Libertad, Peru; Hospital de Alta Complejidad Virgen de la Puerta, Trujillo, La Libertad, Peru; Universidad Cesar Vallejo, Trujillo, La Libertad, Peru; Michigan State University, East Lansing, Michigan

## Abstract

**Background:**

COVID-19 has infected over 4.5 million and killed more than 222,000 people in Peru. Research on key factors for in-hospital mortality in these patients is limited. Our study aims to understand prognostic factors linked to the mortality of patients with COVID-19 receiving treatment in Peru. A modified SHapley Additive exPlanations (SHAP) method was proposed.

Table 1

**Figure:**
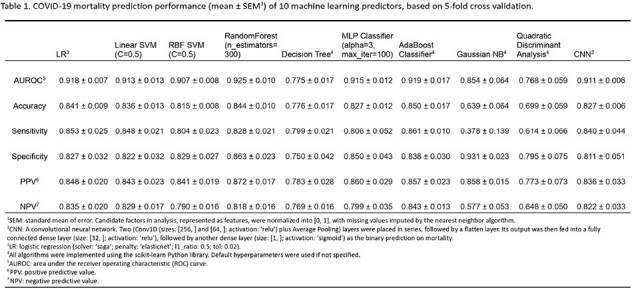

**Methods:**

Clinical and laboratory data were collected prospectively about all patients that were admitted in the inpatient setting at Hospital De Alta Complejidad Virgen De La Puerta and Hospital víctor Lazarte Echegaray Essalud from March 1st to December 31st 2020 with a COVID-19 diagnosis clinically. Ten base predictors were first implemented using the ML algorithms such as Random Forest. Prediction performance on the area under the receiver operating characteristic (AUC) was computed. The SHAP method was then applied to each predictor based on resampled train sets for 30 trials. Factor importance in each trial was estimated by averaging SHAP scores across all predictors, weighted by AUC.

Table 2 (Part 1/3)

**Figure:**
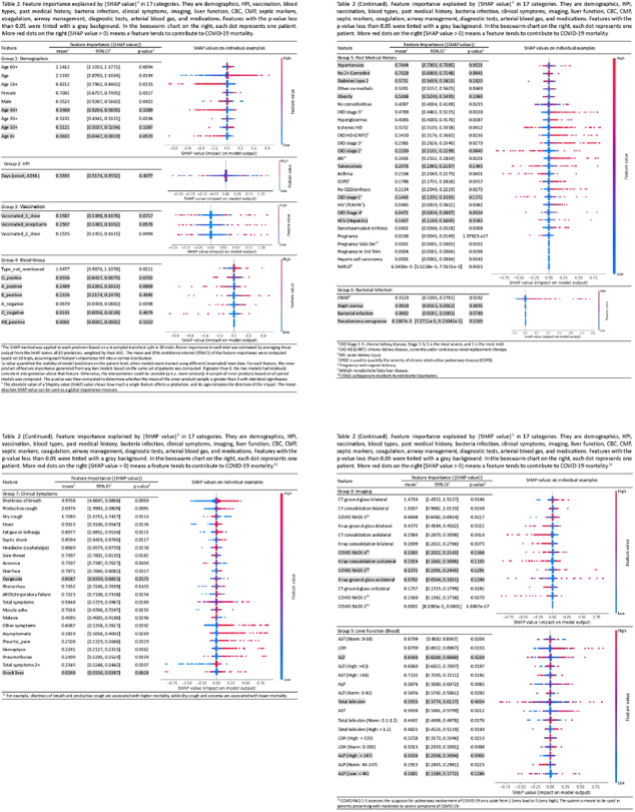

**Results:**

After applying inclusion and exclusion criteria (n=) 1,857 patients were selected from a total of 2,000 admitted patients. Several factors out of 281 in total achieved stable importance scores with statistical significance while others were not. Among poor prognostic factors were low PaO2/FiO2 ratio (SV: 4.135, 95% CI: [4.107, 4.163]), shortness of breath (SV: 4.975, 95% CI: [4.864, 5.086]), and productive cough (SV: 2.037, 95% CI: [1.99, 2.080]). Among favorable factors were high PaO2/FiO2 ratio (SV: 3.832, 95% CI: [3.816, 3.848]), low D dimer (SV: 0.875, 95% CI: [0.859, 0.891]) and lymphocyte counts (SV: 1.319, 95% CI: [1.297, 1.340]). For medications, dexamethasone w. air (SV: 2.186, [2.095, 2.277]) was the most effective. For oxygen support, binasal cannula (SV: 0.841, 95% CI: [0.826, 0.857]) and high flow cannula (SV: 0.663, 95% CI: [0.633, 0.693]) are associated with more favorable prognosis than invasive ones.

Table 2 (Part 2/3)

**Figure:**
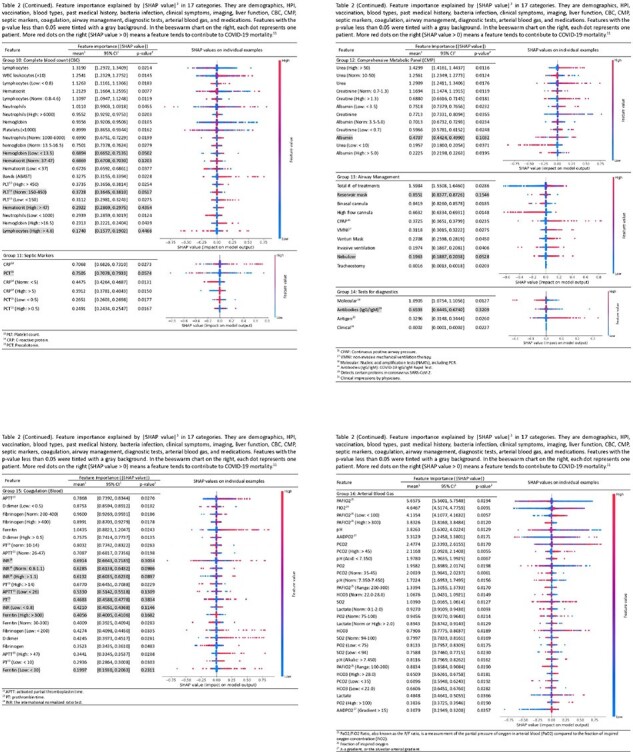

**Conclusion:**

Clinical symptoms and evidence associating acute respiratory distress and sepsis, as well as immune responses less specific to viruses, suggest a likelihood of COVID-19 mortality, consistent with global findings. Our SHAP variant generated reasonable prognostic interpretation about COVID-19 mortality in Peru.

Table 2 (Part 3/3)

**Figure:**
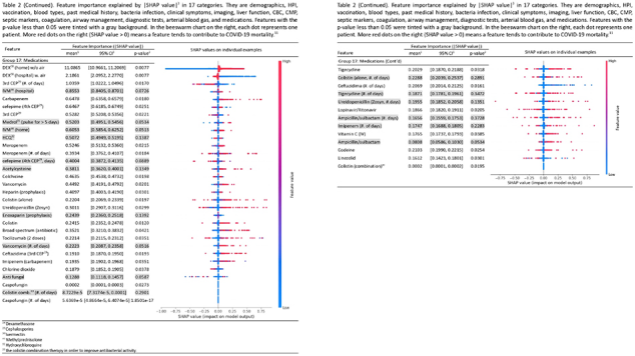

**Disclosures:**

**All Authors**: No reported disclosures

